# Identification of HMGCR as the anticancer target of physapubenolide against melanoma cells by in silico target prediction

**DOI:** 10.1038/s41401-021-00745-x

**Published:** 2021-09-29

**Authors:** Hai-yan Wang, Pian Yu, Xi-sha Chen, Hui Wei, Shi-jie Cao, Meng Zhang, Yi Zhang, Yong-guang Tao, Dong-sheng Cao, Feng Qiu, Yan Cheng

**Affiliations:** 1 Department of Pharmacy, The Second Xiangya Hospital, Central South University, Changsha, 410011 China; 2 Hunan Provincial Engineering Research Centre of Translational Medicine and Innovative Drug, Changsha, 410011 China; 3 Xiangya School of Pharmaceutical Sciences, Central South University, Changsha, 410008 China; 4 School of Chinese Materia Medica and Tianjin State Key Laboratory of Modern Chinese Medicine, Tianjin University of Traditional Chinese Medicine, Tianjin, 301617 China; 5 Department of Pharmacology, College of Pharmaceutical Sciences, Soochow University, Suzhou, 215031 China; 6 Key laboratory of Carcinogenesis and Cancer Invasion, Ministry of Education, Department of Pathology, Xiangya Hospital, School of Basic Medicine, Central South University, Changsha, 410078 China; 7 NHC Key laboratory of Carcinogenesis, Cancer Research Institute, Central South University, Changsha, 410078 China

**Keywords:** physapubenolide, melanoma cells, HMGCR, vemurafenib, drug resistance

## Abstract

Physapubenolide (PB), a withanolide-type compound extracted from the traditional herb *Physalis minima* L., has been demonstrated to exert remarkable cytotoxicity against cancer cells; however, its molecular mechanisms are still unclear. In this study, we demonstrated that PB inhibited cell proliferation and migration in melanoma cells by inducing cell apoptosis. The anticancer activity of PB was further verified in a melanoma xenograft model. To explore the mechanism underlying the anticancer effects of PB, we carried out an in silico target prediction study, which combined three approaches (chemical similarity searching, quantitative structure-activity relationship (QSAR), and molecular docking) to identify the targets of PB, and found that PB likely targets 3-hydroxy-methylglutaryl CoA reductase (HMGCR), the rate-limiting enzyme of the mevalonate pathway, which promotes cancer cell proliferation, migration, and metastasis. We further demonstrated that PB interacted with HMGCR, decreased its protein expression and inhibited the HMGCR/YAP pathway in melanoma cells. In addition, we found that PB could restore vemurafenib sensitivity in vemurafenib-resistant A-375 cells, which was correlated with the downregulation of HMGCR. In conclusion, we demonstrate that PB elicits anticancer action and enhances sensitivity to vemurafenib by targeting HMGCR.

## Introduction

Melanoma is a considerably malignant skin cancer with a low survival rate [1]. A majority of drugs approved for melanoma treatment have mitigated effectiveness owing to acquired resistance [2, 3]. Therefore, it is urgent to develop novel anticancer agents for melanoma treatment. Natural products are extraordinarily valuable resources for the discovery of new drugs and play significant roles in the treatment of cancer [4, 5]. The screening of bioactive compounds will become a potential effective tactic for cancer treatment, including melanoma.

*Physalis minima* L (Solanaceae), commonly known as hairy groundcherry or Deng-Long-Cao, is a medicinal plant that has been used for the treatment of various diseases, such as sore throat, cough, and spleen disorders [6, 7]. Withanolides, a class of steroid derivatives extracted from *Physalis minima* L., have attracted significant attention due to having multiple bioactivities, such as anti-inflammatory, antitumor, and antimicrobial activities [8, 9]. Physapubenolide (PB), a withanolide compound isolated from *Physalis minima* L, has been reported to induce apoptosis and decrease the level of glycolysis through the Akt/p53 pathway in hepatocellular carcinoma cells and trigger apoptosis and autophagy by downregulating TIGAR in breast cancer cells [10, 11]. However, the effects of PB on melanoma cells and the underlying molecular mechanisms remain unclear. Therefore, investigation of the anticancer molecular mechanisms induced by PB could potentially lead to the development of this compound as a novel anticancer agent.

The mevalonate pathway is a pivotal pathway because the generated isoprenoids play vital roles in regulating protein posttranslational modifications such as prenylation and cholesterol synthesis [12–14]. For example, the RAS-mediated signal transduction pathway is activated after the prenylation of RAS, thus promoting tumor cell survival, proliferation, migration, and metastasis [15, 16]. 3-Hydroxy-methylglutaryl CoA reductase (HMGCR), a main enzyme that catalyzes the conversion of HMG-CoA to mevalonate, plays an indispensable role in the mevalonate pathway [17, 18]. Previous studies have shown that HMGCR was upregulated in multiple tumors, including breast cancer, bladder cancer, glioblastoma, and esophageal squamous cell carcinoma, and promoted cancer cell proliferation, migration, and metastasis [19, 20]. Therefore, HMGCR is a promising therapeutic target for cancer treatment.

In this study, we show that PB exhibits potent anticancer activity in melanoma cells by inducing cell apoptosis, thus inhibiting cell proliferation and migration. Furthermore, we identified the target of PB by a combinatorial target prediction strategy and demonstrated that PB interacted with HMGCR and decreased HMGCR protein expression. Additionally, we found that PB reverses vemurafenib resistance by decreasing HMGCR protein expression. Our study not only reveals the anticancer activity of PB in melanoma cells and the underlying mechanism but also provides a potential strategy to enhance the sensitivity of melanoma cells to vemurafenib.

## Materials and methods

### Cell lines and culture

Human melanoma cells (SK-MEL-5, A-375, and vemurafenib-resistant A-375), human breast cancer cells (MCF-7 and MDA-MB-231), and human normal cells (HaCaT and MCF-10A) were cultured in Dulbecco’s modified Eagle’s medium (DMEM)/high glucose medium supplemented with 10% fetal bovine serum (ExCell Bio). Cells were maintained at 37 °C in a humidified incubator with 5% CO_2_.

### Reagents and antibodies

Antibodies against cleaved caspase-3 (#9661, dilution: 1:1000), YAP (#14074, dilution: 1:1000) and p-YAP (Ser 127) (#13008, dilution: 1:1000) were purchased from Cell Signaling Technology (Danvers, MA, USA). Antibodies against HMGCR were purchased from Abcam (ab174830, dilution: 1:1000) and Proteintech (13533-1-AP, dilution: 1:50). Antibodies against N-cadherin (22018-1-AP, dilution: 1:1000), β-catenin (17565-1-AP, dilution: 1:1000), and β-actin (20536-1-AP, dilution: 1:1000) were purchased from Proteintech.

### Cell viability assay

Cells (5 × 10^3^ cells/well) were plated in 96-well culture plates and treated with various concentrations of PB for the indicated length of time. Then, 10 μL of CCK8 reagent (Biotool) was added to each well and incubated for 1 h. At the end of this incubation, the absorbance at 450 nm was measured.

### siRNA transfection

siRNAs targeting HMGCR were purchased from RiboBio (Ribo Biotechnologies, Guangzhou, China). For siRNA transfection, vemurafenib-resistant A-375 cells were seeded in 6-well culture plates. siRNAs (100 nM) were transfected into cells using Lipofectamine 2000 reagent according to the manufacturer’s recommendations. Then, cells were seeded in 96-well culture plates and exposed to media with or without various concentrations of vemurafenib.

### shRNA transfection

A-375 and SK-MEL-5 cells were seeded in 6-well plates. When the cell densities reached 60% confluence, 1 mL of the lentiviral supernatant containing the lentiviral construct for sh-HMGCR plasmid or vector plasmid was added to the cells. After 24 h of infection, 1 mL of the lentiviral supernatant was added. After 48 h, culture medium containing 0.5 µg/mL puromycin was added to the cells for 3 days. After 3 days, Western blotting was performed to validate the HMGCR knockdown effect in these cells.

### Western blot analysis

After treatment with PB, the cells were washed with PBS and lysed in RIPA buffer (Beyotime, Haimen, China) supplemented with a protease inhibitor cocktail and phosphatase inhibitor cocktail (Selleck). Protein concentrations were measured using a G250 protein assay kit (Beyotime, Haimen, China). Proteins were separated by 12% SDS-PAGE and transferred to a PVDF membrane. Next, the membranes were blocked with 10% skim milk and then probed with primary antibodies and peroxidase-conjugated secondary antibodies. Subsequently, membranes were visualized with an enhanced chemiluminescent detection kit.

### EdU assay

Proliferating cells were stained with EdU using the Cell Light EdU DNA Cell Proliferation Kit (RiboBio Co, Guangzhou, China). Cells were seeded in 96-well culture plates and treated with 0.5 μM or 1 μM PB for 48 h. Then, the cells were treated with 50 μM EdU for 4 h at 37 °C. After being fixed with 4% paraformaldehyde for 15 min, cells were treated with 0.5% Triton X-100 for 20 min and rinsed with PBS three times. Thereafter, cells were exposed to 100 μL of 1×Apollo^®^ reaction cocktail for 30 min and incubated with 5 μg/mL Hoechst 33342 to stain the cell nuclei for 30 min. Images were captured using a fluorescence microscope.

### Apoptosis assay

Cell apoptosis was determined by flow cytometric analysis of Annexin V-FITC/PI double-staining. After treatment with 2 μM or 4 μM PB for 48 h, cells were harvested and rinsed with cold PBS twice by centrifugation at 1000 × *g* and resuspended in 100 μL of binding buffer (BD cat. no. 556547). Next, 5 μL of Annexin-V (BD cat. no. 556420) and 5 μL of PI (Propidium Iodide) (BD cat. no. 556463) were added to the solution and incubated for 15 min at room temperature in the dark for flow cytometric (Becton Dickison) analysis.

### Colony formation assay

Cells (1000 cells/well) were seeded in 6-well culture plates for 24 h and then treated with 0.5 μM or 1 μM PB for 24 h. Cells were cultured at 37 °C for 14 days with medium changes every third day. Cells were fixed with paraformaldehyde and stained with 0.5% crystal violet, and colonies were counted under a microscope.

### Cell migration assay

Cells were cultured in 6-well plates until the cell densities reached 80% confluence. A horizontal scratch was created using a sterile 10 µL pipette tip. Cells were washed with PBS to remove cell debris and treated with 2 μM or 4 μM PB. Three different areas in each dish were selected to compare the distance that cells had migrated from the scratch border. Images were captured at 0, 24, and 48 h to assess the extent of cell migration.

### Xenograft mouse model

Xenograft tumors were established by subcutaneous injection of 5 × 10^6^ A-375 cells in 0.1 mL of PBS into the right front legs of BALB/c mice (mean age of 5 weeks). After 12–14 days, the tumor sizes were measured using micrometer calipers, and then the mice were randomly divided into 3 groups (9 nude mice/group). Then, saline, 5 mg/kg PB, or 10 mg/kg PB were intraperitoneally injected for 10 days. Tumor volumes were measured at 2-day intervals to generate a tumor growth curve. Tumor volume (TV) was estimated by the following formula: TV (mm^3^) = *D*/2 × *d*^2^, where *D* is the length of the tumor and *d* is the width of the tumor. After 20 days, the mice were sacrificed, and the tumors were removed and weighed.

### Immunohistochemical staining

Formalin-fixed tissue samples were embedded in paraffin, and then the paraffin-embedded specimens were cut into serial sections (4-mm thick). The Ki67 and HMGCR assays were performed according to the manufacturer’s recommendations. This study was carried out in accordance with the local guidelines for the care of laboratory animals of the Animal Experimental Center and was approved by the Ethics Committee For Research On Laboratory Animals and Use of The Central South University.

### Ligand-based prediction approach

To identify the potential targets of PB to explore its antitumor mechanism, we applied multiple in silico target prediction approaches to implement target prediction and screening in a layer-by-layer manner. Herein, to quickly narrow the target space of PB, four commonly used target prediction platforms were aggregated as the first step, including PPB2, Swiss TargetPrediction, HitPickV2 and ChEMBL [21–24]. For each prediction platform, the top 15 predicted target proteins were screened and processed first according to the respective rules of the platform. Subsequently, to aggregate the target proteins retrieved from different platforms into a target list, all target IDs were uniformly converted into UniProt Accession.

To validate the screened target proteins, Gene Ontology (GO) and Kyoto Encyclopedia of Genes and Genomes (KEGG) functional analyses were performed through the Diversity Visualization Integrated Database (DAVID 6.8, https://david.ncifcrf.gov/). Subsequently, the obtained GO terms and KEGG pathways with *P* values < 0.01 were selected to further confirm whether these screened proteins focused on the major pharmacological activities exerted by PB. Finally, the reliable target proteins predicted by at least two target prediction tools were selected for further screening.

### Consensus SAR modeling

To further screen the key target proteins for the anticancer effects exerted by PB, consensus SAR models based on machine learning were subsequently built for the target proteins screened before. First, the experimental quantitative activity data for each target protein were collected from the BindingDB database. The activity data for each target protein were filtered to keep only activity endpoints with half-maximum inhibitory concentration (IC_50_), half-maximum effective concentration (EC_50_) or equilibrium inhibition constant (*K*_i_). Then, molecular structures in multiple formats (e.g., mol, sdf) were converted to canonical SMILES uniformly using OpenBabel (version 2.4.1) and duplicated molecules were then eliminated based on InChIKey strings. For each protein, to avoid the incline deviation result from the weighty molecular scaffolds, scaffold analysis was carried out using molecular operating environment (MOE, version 2018) software to randomly remove redundant molecules with the same scaffolds before SAR modeling.

For each protein, the molecule was considered an active sample if the mean activity value was below 10 µM; otherwise, it was considered an inactive sample. According to this criterion, each target dataset was split into a positive set and a negative set. Then, a series of SAR models were built separately based on random forest (RF) and three types of molecular descriptors (CATS, MACCS and MOE2d) and validated by 5-fold cross validation using the KNIME framework (version 3.7.2). Herein, the molecular descriptors were all calculated by MOE and ChemoPy software (Descriptor dimension, CATS: 210, MACCS: 166 and MOE2d: 206). To improve the prediction ability, three SAR models based on diverse descriptors for each protein were aggregated into the consensus SAR model with an averaged output. Eventually, the commonly used evaluation metrics were applied to effectively evaluate the generalization ability of those models, including sensitivity (SE), specificity (SP), accuracy (ACC), and the area under the curve value of the receiver operating characteristic curve (AUC).

### Molecular docking

To assist in verifying the target proteins screened before, molecular docking was implemented to further simulate the binding affinity between PB and the target proteins with three-dimensional crystal structures. The principle of molecular docking is to calculate optimal binding geometries and energies for prediction mainly by positioning the ligand in different orientations and conformations within the binding site. In this part, X-ray crystal structures of screened target proteins were first downloaded from Protein Data Bank (PDB) and then prepared through MOE to remove crystallographic water molecules and ions, add fixed hydrogens and assign protonation states, etc. The native ligand in each crystal structure was used to define the binding site. Molecular docking with the induced fit mode was then carried out to explore the interaction between PB and the target proteins. During this step, 30 poses of each ligand were generated, and the other parameters were set to the default values. Finally, the optimal structural conformation was determined according to the score values. As a control, the typical ligands were docked with corresponding target proteins to assist in verifying potential targets.

### Statistical analysis

GraphPad Prism 5 was utilized to carry out statistics analysis. Data were presented as the mean±SEM. Using Student’s *t*-test (two tailed) analysis of variance for analyzing the difference between groups. Levels of *P* < 0.05 were considered statistically significant.

## Results

### PB significantly inhibits proliferation and induces apoptosis in melanoma cells

**Fig. 1:**
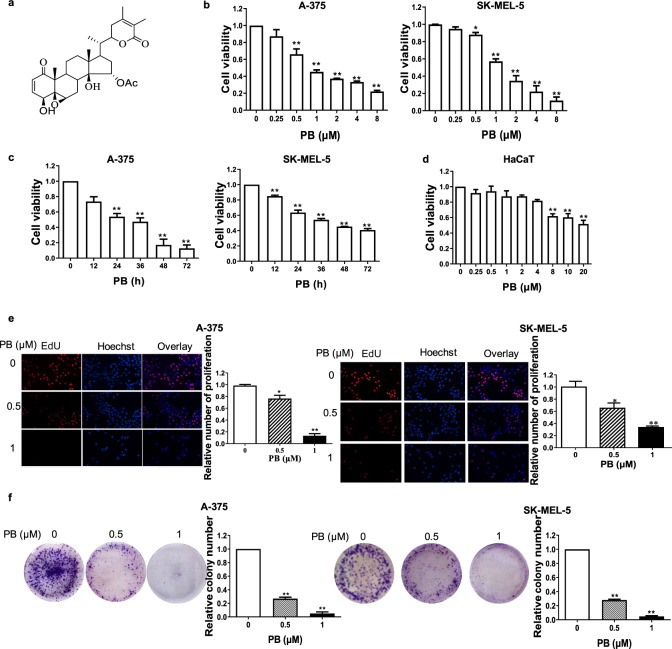
PB significantly inhibits the proliferation of melanoma cells. **a** The chemical structure of the small molecule compound PB. **b**–**c** A-375 and SK-MEL-5 cells were treated with the indicated concentrations of PB for 72 h or with 2 μM PB for different time periods. Cell viability was determined with the CCK8 reagent. **d** HaCaT cells were treated with the indicated concentrations of PB for 72 h, and cell viability was determined with the CCK8 reagent. **e** A-375 and SK-MEL-5 cells were treated with 0.5 μM or 1 μM PB for 48 h. EdU-labeled replicating cells were examined under a fluorescence microscope. Red and blue cells were counted in a blind manner. **f** A-375 and SK-MEL-5 cells were treated with 0.5 μM or 1 μM PB and detected with a colony formation assay. Data are shown as the mean ± SEM of three independent experiments. *P < 0.05, **P < 0.01 vs. the control group.

We first measured the effects of PB (structure shown in Fig. 1a) on cell viability by CCK8 assay and found that PB inhibited cell proliferation in a dose- and time-dependent manner (Fig. 1b, c), with IC_50_ values (72 h) of 1.225 and 1.416 μM in A-375 and SK-MEL-5 cells, respectively. Importantly, there was no significant cytotoxicity against normal human keratinocytes (HaCaT cells) in response to PB at low concentrations, and PB inhibited cell proliferation at high concentrations (Fig. 1d). The EdU incorporation assay further demonstrated that PB inhibited the proliferation of melanoma cells, as indicated by the decreased number of EdU-positive cells (Fig. 1e). To examine the long-term effects of PB on cell growth, a colony formation assay was performed. The colony-forming ability of melanoma cells decreased in a dose-dependent manner in PB-treated cells (Fig. 1f). These results demonstrated that PB selectively shows significant cytotoxic effects against melanoma cells.

**Fig. 2:**
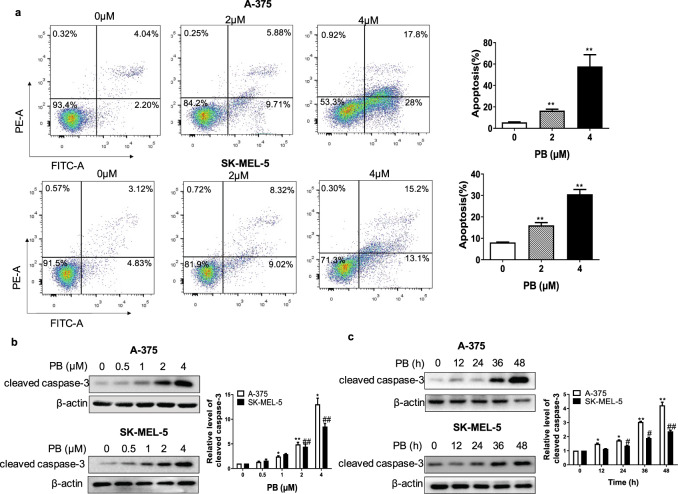
PB causes apoptosis in melanoma cells. **a** A-375 and SK-MEL-5 cells were stained with annexin V and PI and analyzed by flow cytometry. **b**–**c** A-375 and SK-MEL-5 cells were treated with the indicated concentrations of PB for 24 h or with 2 μM PB at different time points. Western blotting analysis was applied to detect the expression levels of cleaved caspase-3. β-actin served as a loading control. Data are shown as the mean ± SEM of three independent experiments. *P < 0.05, **P < 0.01, ^#^*P* < 0.05, ^##^*P* < 0.01 vs. the control group.

We next examined the effects of PB on apoptosis in A-375 and SK-MEL-5 cells. Annexin V-FITC/PI flow cytometry analysis was utilized to evaluate the induction of apoptosis in cells treated with the indicated concentrations of PB. Fig. 2a shows that PB triggered apoptosis in a dose-dependent manner, as manifested by an increase in annexin V staining. Additionally, we examined the expression of the apoptosis-associated protein cleaved caspase-3. As shown in Fig. 2b, c, cleaved caspase-3 expression increased after PB treatment in a dose- and time-dependent manner. These results demonstrate that PB effectively induces apoptosis in melanoma cells.

### PB inhibits the migration of melanoma cells

**Fig. 3:**
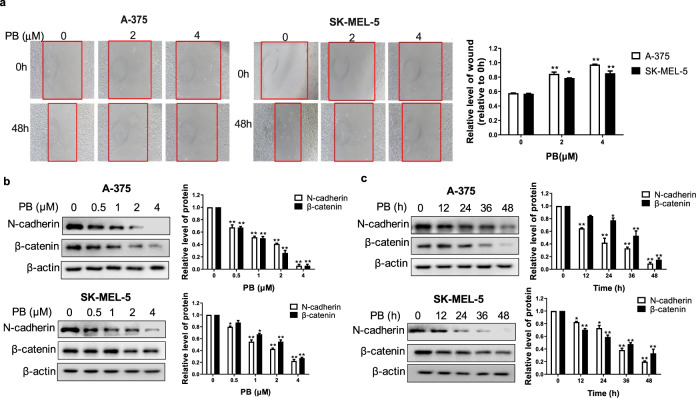
PB effectively inhibits the migration of melanoma cells. **a** A-375 and SK-MEL-5 cells were treated with 2 μM or 4 μM PB, and subjected to cell migration assays, the red line indicates the edge of migrating cells at a given time point. **b**–**c** A-375 and SK-MEL-5 cells were treated with the indicated concentrations of PB for 24 h, or were treated with 2 μM PB for different time periods. Western blotting analysis was applied to detect the expression levels of the N-cadherin and β-catenin. β-actin served as a loading control. Data are shown as the mean ± SEM of three independent experiments. *P < 0.05, **P < 0.01 vs. the control group.

We further examined whether PB can affect the migration of tumor cells. Cell migration was assessed with a wound-healing assay. As shown in Fig. 3a, PB decreased the migration of melanoma cells in a concentration-dependent manner. Epithelial–mesenchymal transition (EMT) is a pivotal process in the migration and invasion of malignant tumors and is characterized by the acquisition of mesenchymal markers such as N-cadherin [25, 26]. β-catenin, a main regulator of cell–cell adhesion, is also a critical indicator of the migration of cancer cells [27]. Western blotting showed that the expression levels of N-cadherin and β-catenin decreased in a dose- and time-dependent manner in cells treated with PB (Fig. 3b, c). These results demonstrate that PB not only inhibits cell proliferation but also suppresses the migration of melanoma cells.

### Identification of the targets of PB by combinatorial target prediction

**Fig. 4:**
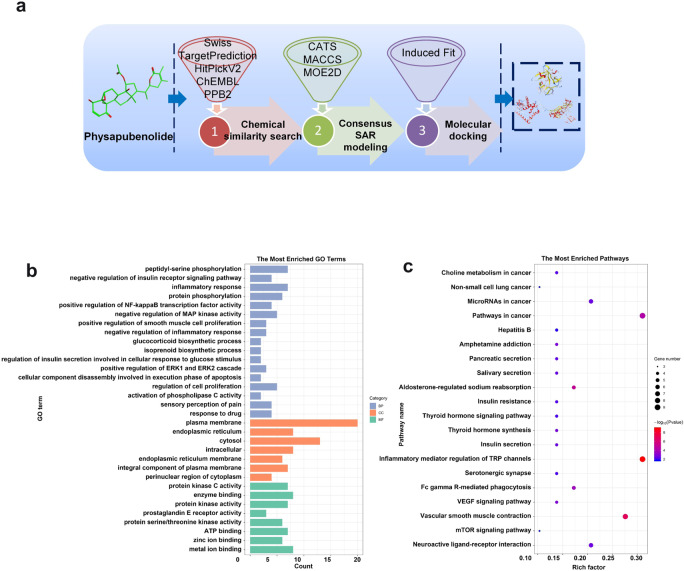
A combinatorial target prediction strategy identifies potential targets of PB. **a** A combinatorial target prediction strategy to screen the potential target proteins of PB stepwise. **b**–**c** The top enriched GO terms and KEGG pathway analysis to validate the target protein effects.

To elucidate the mechanism through which PB inhibits cancer cell proliferation and migration, we performed a combinatorial target prediction strategy for the stepwise screening of the potential target proteins of PB (Fig. 4a). First, chemical similarity searching was performed to preliminarily screen potential target proteins. Four ligand-based prediction platforms, Swiss TargetPrediction, HitPickV2, ChEMBL and PPB2, were first used to narrow the target space of the query molecule PB. After target prediction and aggregation, 37 target proteins in total were obtained. These target proteins were validated to be closely related to hyperlipemia, cardiac disease, cancer, etc., which is consistent with pharmacological activities of PB according to the top enriched GO terms and KEGG pathways (Fig. 4b, c), such as pathways in cancer (hsa05200), aldosterone-regulated sodium reabsorption (hsa04960), insulin secretion (hsa04911), regulation of cell proliferation (GO:0042127), negative regulation of insulin receptor signaling pathway (GO:0046627), and sensory perception of pain (GO:0019233).

**Fig. 5:**
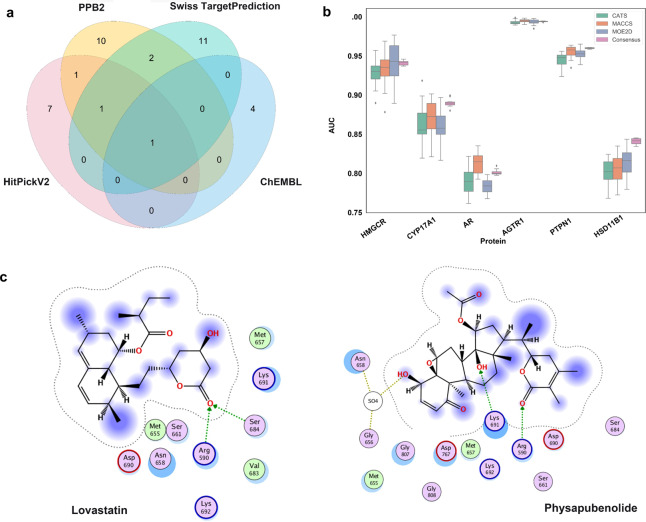
Performance evaluation and prediction results of different target-prediction methods. **a** Overlapping target proteins predicted by diverse target prediction platforms. **b** AUC scores of the consensus SAR models and single SAR models based on three descriptors. **c** The optimal structural conformation of HMGCR and the ligands lovastatin and PB.

A Venn diagram was then constructed to observe the overlay. Finally, 6 proteins that were predicted by at least two platforms were screened as potential targets to study the anticancer effects exerted by PB, including 3-hydroxy-3-methylglutaryl-coenzyme A reductase (HMGCR), the steroid 17-alpha-hydroxylase/17,20 lyase (CYP17A1), the androgen receptor (AR), type-1 angiotensin II receptor (AGTR1), corticosteroid 11-β-dehydrogenase isozyme 1 (HSD11B1) and tyrosine-protein phosphatase nonreceptor type 1 (PTPN1) (Fig. 5a).

Consensus SAR modeling was then applied to implement a targeted study for the screened target proteins. The chosen evaluation metric, AUC, values were all basically above 0.70, while the ACC, SE, and SP values also presented the same trend (Supplementary Fig. 1). These data are all strong evidence that these constructed SAR models have good predictive ability. For each protein, consensus SAR models with averaged output were aggregated from three single models based on different descriptors to improve model performance. Compared with single models based on each molecular descriptor, the statistical evaluation results of the consensus SAR models presented reduced variability and fluctuation (Fig. 5b). Thus, we confirmed that the consensus SAR models have been shown to be more robust and practical for subsequent prediction compared with single models. Eventually, the prediction results by consensus SAR modeling selected HMGCR as a potential target of PB (Supplementary Table 1). To further demonstrate the reliability of HMGCR as a PB target, molecular docking between HMGCR and PB and lovastatin (HMGCR inhibitor) was performed to further observe their binding. As shown in Fig. 5c, the main binding force of both lovastatin and PB is hydrogen bonding; additionally, the score value for PB was −8.7 while that for lovastatin was −7.5. Therefore, we can infer that PB indeed has a certain degree of interaction with the target protein HMGCR. In addition, it has been demonstrated that HMGCR plays crucial roles in the development of tumors, such as promoting the proliferation, migration and invasion of tumor cells vv; thus, HMGCR was finally chosen as a potential target of PB to exert antitumor effects for in-depth research.

### PB exerts anticancer activity by inhibiting the HMGCR-mediated signaling pathway

**Fig. 6:**
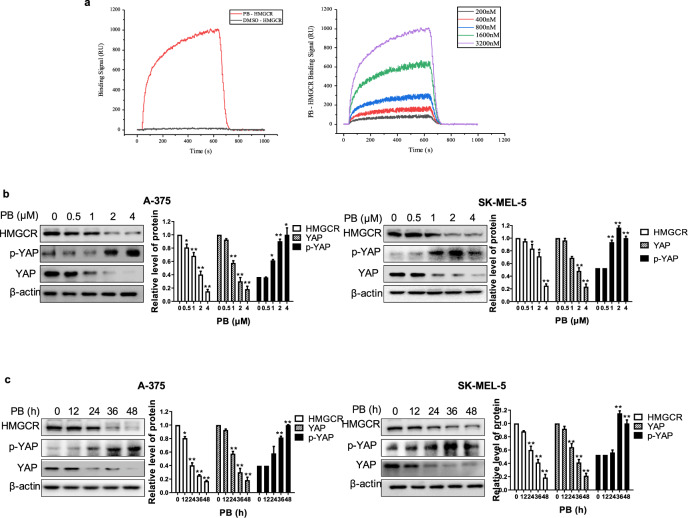
PB exerts anticancer activity by inhibiting the HMGCR-mediated pathway. **a** The interaction between PB and HMGCR was determined by SPR assay. **b**–**c** A-375 and SK-MEL-5 cells were treated with the indicated concentrations of PB for 24 h or with 2 μM PB for different lengths of time. Western blotting analysis was applied to detect the expression levels of HMGCR, YAP and p-YAP. β-actin served as a loading control. Data are shown as the mean ± SEM of three independent experiments. *P < 0.05, **P < 0.01 vs. the control group.

We next sought to determine whether there was an interaction between PB and HMGCR. SPR-based direct binding assays showed that PB had a strong binding affinity to the HMGCR protein and that the binding signal became stronger as the concentration increased (Fig. 6a). The kinetic and affinity parameters between PB and HMGCR binding are shown in Table 1. Furthermore, we found that PB decreased the protein expression of HMGCR in a dose- and time-dependent manner in melanoma cells (Fig. 6b, c). Additionally, we detected the protein expression of YAP, a downstream player of HMGCR [28]. It has been reported that inhibiting HMGCR causes a prominent accumulation of phosphorylated YAP (p-YAP) and reduced YAP protein expression [29]. As shown in Fig. 6b, c, there was a decrease in YAP expression but an increase in p-YAP in PB-treated melanoma cells in a dose- and time-dependent manner. These results suggest that the HMGCR-mediated signaling pathway contributes to the anticancer effects of PB in melanoma cells. Furthermore, knockdown of HMGCR did not affect PB-induced cell death compared with PB treatment alone (Supplementary Fig. 2), indicating that PB exerts its antitumor activity by targeting HMGCR.

**Table 1:** Kinetic and affinity parameters between PB and HMGCR.

Phase	Stationary phase	Avg kon (1/Ms)	Avg koff (1/s)	Avg *K*_D_ (M)	Int. Intensity
HMGCR	PB	6.06 × 10^2^	8.83 × 10^−4^	1.46 × 10^−6^	Strong

We further compared the antitumor activity of PB and lovastatin (a competitive inhibitor of HMGCR) and found that PB shows stronger cytotoxicity than lovastatin in A-375 and SK-MEL-5 cells (Supplementary Fig. 3), which is consistent with the molecular docking results, showing that PB has stronger binding to HMGCR than lovastatin (Fig. 5c). These results suggest that PB exerts its anticancer activity by inhibiting the HMGCR-mediated signaling pathway.

### PB suppresses melanoma cell proliferation and reduces HMGCR expression in vivo

**Fig. 7:**
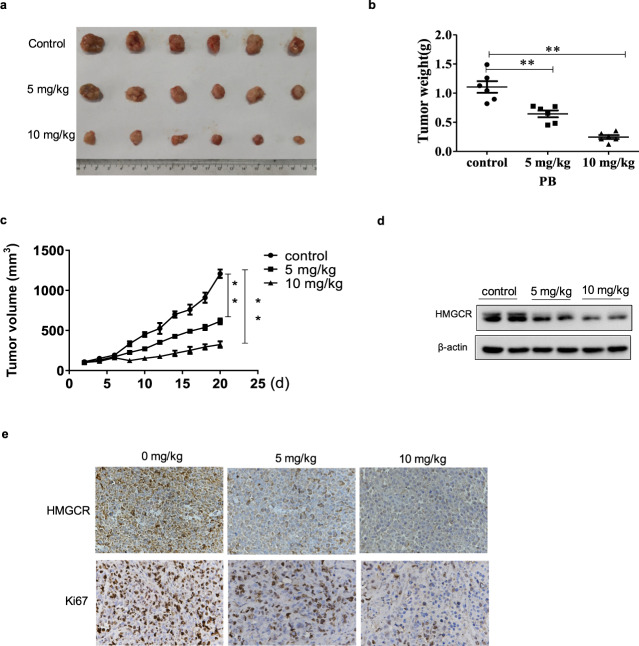
PB inhibits melanoma cell proliferation in vivo. When subcutaneous A-375 tumor xenografts reached 80–100 mm^3^, mice were injected intraperitoneally with saline, 5 mg/kg PB, 10 mg/kg PB for 10 days. **a** Dissected tumors. **b** Tumor weight. **c** Volume changes of tumor. **d** Western blotting analysis of the expressions of HMGCR in tumor samples, β-actin served as a loading control. **e** Immunohistochemical staining for Ki67 and HMGCR in tumor tissues.

To further determine the antitumor effects of PB, an A-375 xenograft model was established. PB significantly inhibited tumor growth, as demonstrated by the significantly reduced tumor volumes and weights compared to the control group (Fig. 7a–c). Additionally, the Ki67 assay indicated that PB inhibited the proliferation of melanoma cells (Fig. 7e). We further examined the effects of PB on the expression of HMGCR in tumor tissues using Western blot and immunohistochemistry and observed that PB treatment resulted in a significant downregulation of HMGCR compared with the control (Fig. 7d, e). These results suggest that PB suppresses tumor growth in a human A-375 xenograft model by downregulating HMGCR.

### PB restores vemurafenib sensitivity in vemurafenib-resistant A-375 cells by downregulating HMGCR expression

**Fig. 8:**
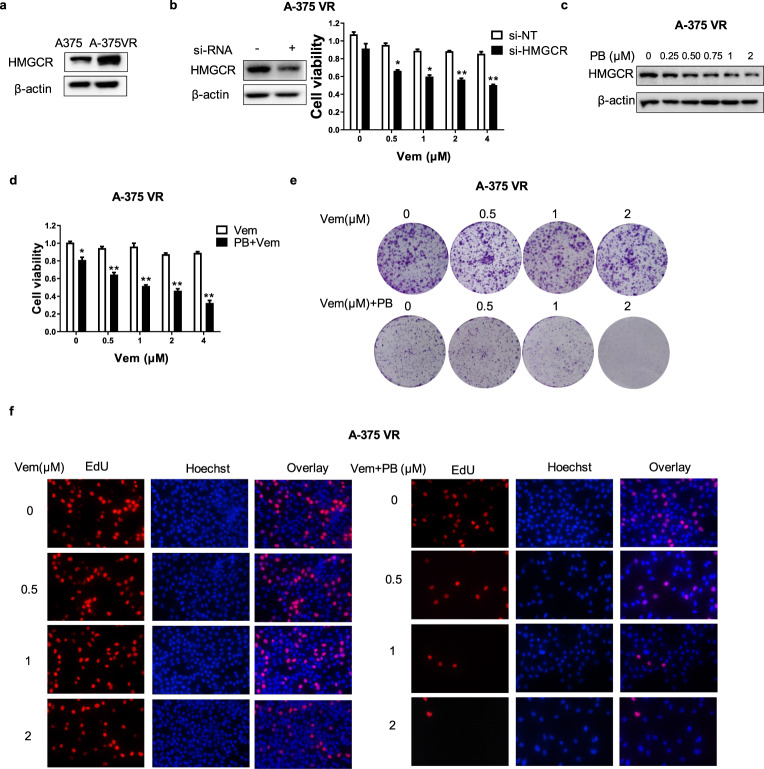
PB enhances the sensitivity of vemurafenib-resistant cells by downregulating HMGCR. **a** The levels of HMGCR in A-375 and vemurafenib-resistant A-375 cells were measured by Western blotting. β-actin served as a loading control. **b** Vemurafenib-resistant A-375 cells were transfected with nonspecific RNA and HMGCR siRNA for 72 h. The level of the HMGCR protein was examined by Western blotting. β-actin served as a loading control. Cells were then treated with various concentrations of vemurafenib for 72 h, and their viability was examined by CCK8 assay. **c** Vemurafenib-resistant A-375 cells were treated with the indicated concentrations of PB for 72 h, and Western blotting analysis was utilized to detect the expression levels of HMGCR. β-actin served as a loading control. Vemurafenib-resistant A-375 cells were treated with the indicated concentrations of vemurafenib in combination with 0.75 μM PB and (**d**) the cell viability was examined by CCK8 assay. **e** The clonogenic ability was detected by a colony formation assay. **f** EdU staining was performed, and cells were examined under a fluorescence microscope. The red and blue cells were counted in a blind manner. Data are shown as the mean ± SEM of three independent experiments. *P < 0.05, **P < 0.01 vs. the control group.

We further observed that HMGCR was overexpressed in vemurafenib-resistant cells compared with vemurafenib-sensitive melanoma cells A-375 (Fig. 8a). To demonstrate whether HMGCR is associated with vemurafenib-induced resistance, vemurafenib-resistant A-375 cells transfected with HMGCR siRNA were exposed to various concentrations of vemurafenib, and cell viability was measured. Fig. 8b shows that knockdown of HMGCR significantly decreased the viability of vemurafenib-resistant A-375 cells compared with vemurafenib treatment alone. These results revealed that HMGCR plays an important role in the induction of vemurafenib resistance in melanoma cells. Importantly, PB decreased the protein expression of HMGCR in a dose-dependent manner in vemurafenib-resistant A-375 cells (Fig. 8c). Combination treatment with vemurafenib and PB increased the cytotoxic effects of vemurafenib in resistant cells (Fig. 8d). Additionally, PB combination treatment significantly reduced the colony-forming ability of resistant cells compared with vemurafenib treatment alone (Fig. 8e). An EdU incorporation assay was utilized to further examine the effects of combination treatment, and the results showed that the number of EdU-positive cells decreased in the presence of PB in resistant cells compared with vemurafenib treatment alone (Fig. 8f). These results suggest that PB restores vemurafenib sensitivity in vemurafenib-resistant A-375 cells by decreasing HMGCR.

## Discussion and conclusion

**Fig. 9:**
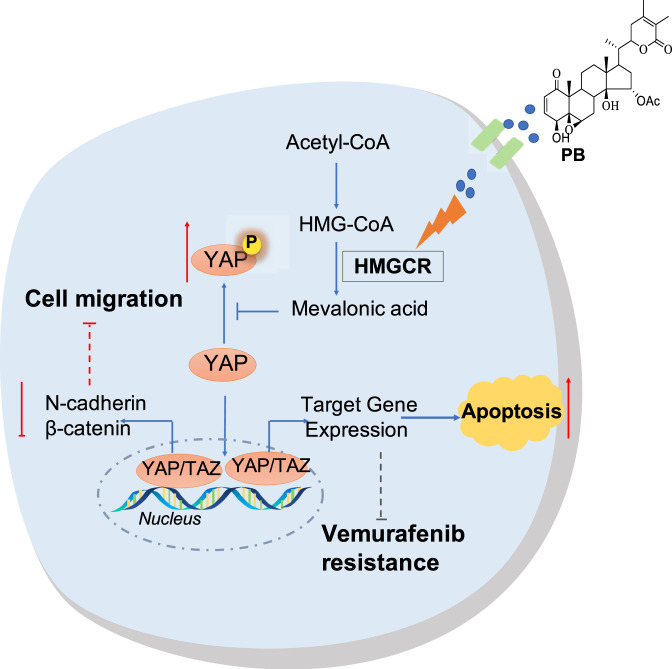
PB inhibits the proliferation and migration of melanoma cells by targeting HMGCR/YAP pathway. PB induced phosphorylation level of YAP but reduced YAP nuclear localization by targeting HMGCR. PB modulate apoptosis, migration, and vemurafenib resistance in melanoma by regulating YAP nuclear localization.

Traditional Chinese medicines have shown good anticancer activity and promising therapeutic value against cancer, but the unclear molecular mechanisms have greatly limited their clinical application for the treatment of cancer. A better understanding of how natural product agents exert antitumor effects could facilitate the development of compounds as anticancer agents. Although the anticancer activity of PB is emerging, the underlying mechanisms and direct targets are unknown. Investigating the related molecular mechanisms induced by PB can potentially lead to novel anticancer agents. In the present study, we demonstrated that PB exhibited significant anticancer activity in melanoma cells in vitro and in vivo. Importantly, we predicted the target of PB by the combinatorial target prediction strategy and identified HMGCR as the potential target of PB (Fig. 9).

HMGCR is the rate-limiting enzyme of the mevalonate pathway. Numerous studies have demonstrated the high expression of HMGCR in many tumors, and inhibiting its activity could affect the functions of tumor cells, such as proliferation and migration [19, 30]. YAP, a main nuclear mediator of the Hippo signaling pathway, acts as a transcriptional coactivator modulating genes involved in differentiation, proliferation, apoptosis and migration [31, 32]. It has been reported that silencing YAP decreases the proliferation of hepatocellular carcinoma (HCC) cells by reducing the activation of ERK and AKT [33]. YAP is also involved in the regulation of cell migration in cancer cells by inducing the transcription of N-cadherin, Twist and β-catenin [19]. Additionally, YAP inhibits apoptosis via the mitochondrial apoptosis pathway [34]. The mevalonate pathway modulates YAP phosphorylation at Ser127, leading to the degradation of YAP [35]. It has been reported that the overexpression of HMGCR promotes YAP/TAZ nuclear localization and activity and decreases the concentration of phosphorylated YAP in the cytoplasm [36]. We found that PB induced an increase in the phosphorylation level of YAP at Ser127 but a decrease in the expression of YAP in a dose- and time-dependent manner in melanoma cells. Our data suggested that PB inhibited the proliferation and migration of melanoma cells by suppressing HMGCR/YAP signaling. In addition, knockdown of HMGCR did not affect PB-induced cell death. Thus, we demonstrated that HMGCR is the anticancer target of PB, and the antitumor activity of PB was associated with the expression of HMGCR in cancer cells.

Vemurafenib, an inhibitor of BRAF^V600E^, is a major therapeutic drug against melanoma [37, 38]. However, acquired resistance often leads to therapy limitations [39, 40]. Therefore, it is of great importance to investigate the mechanisms involved in vemurafenib resistance and develop effective strategies to restore vemurafenib sensitivity in melanoma cells. We found that the level of HMGCR was higher in vemurafenib-resistant cells, and the sensitivity of resistant cells increased after silencing HMGCR, indicating that HMGCR is well correlated with vemurafenib resistance. Furthermore, the combined usage of vemurafenib and PB had a synergetic effect in vemurafenib-resistant melanoma cells, suggesting that combination treatment with PB could reverse vemurafenib resistance by decreasing HMGCR.

Based on these results, we confirm that PB exerts anticancer effects by inhibiting the HMGCR/YAP signaling pathway. Additionally, the combined usage of PB and vemurafenib may be a novel therapeutic tactic to reverse the resistance to vemurafenib. Our study not only uncovers the anticancer activity of PB in melanoma cells and the underlying mechanism but also provides a potential strategy to enhance the sensitivity of vemurafenib in drug-resistant melanoma cells.

## Supplementary information

Supplementary Material
