# Supplementary material for: Identification of HMGCR as the anticancer target of physapubenolide against melanoma cells by in silico target prediction
Source: Acta Pharmacol Sin. 2021 Sep 29;43(6):1594–604. doi: 10.1038/s41401-021-00745-x (PMC9160031; doi:10.1038/s41401-021-00745-x)
Supplement: Supplementary file 1 — Supplementary Material [file 41401_2021_745_MOESM1_ESM.docx]

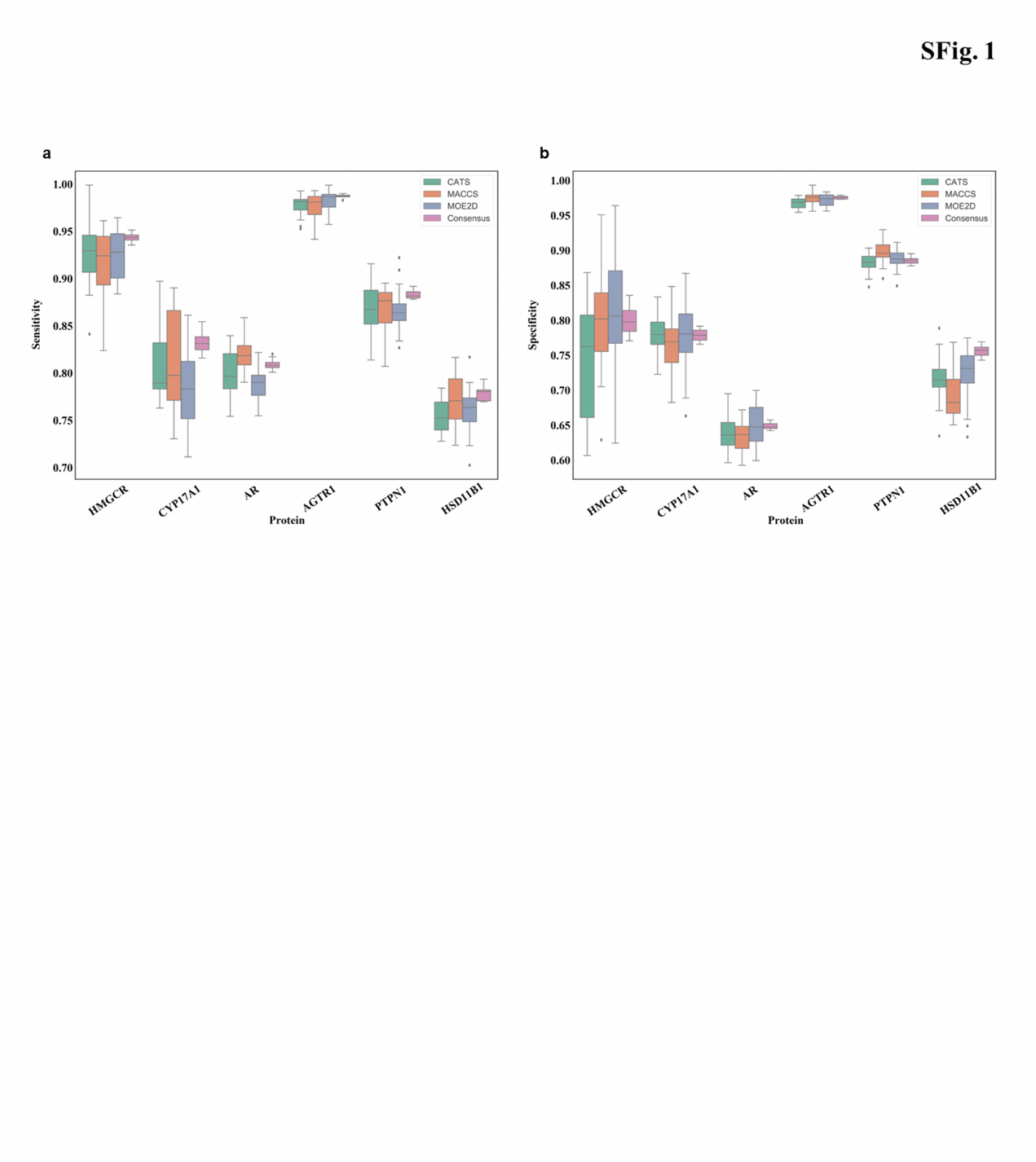


**Supplementary Fig.1**. The evaluation metric AUC for the screened target proteins.

**
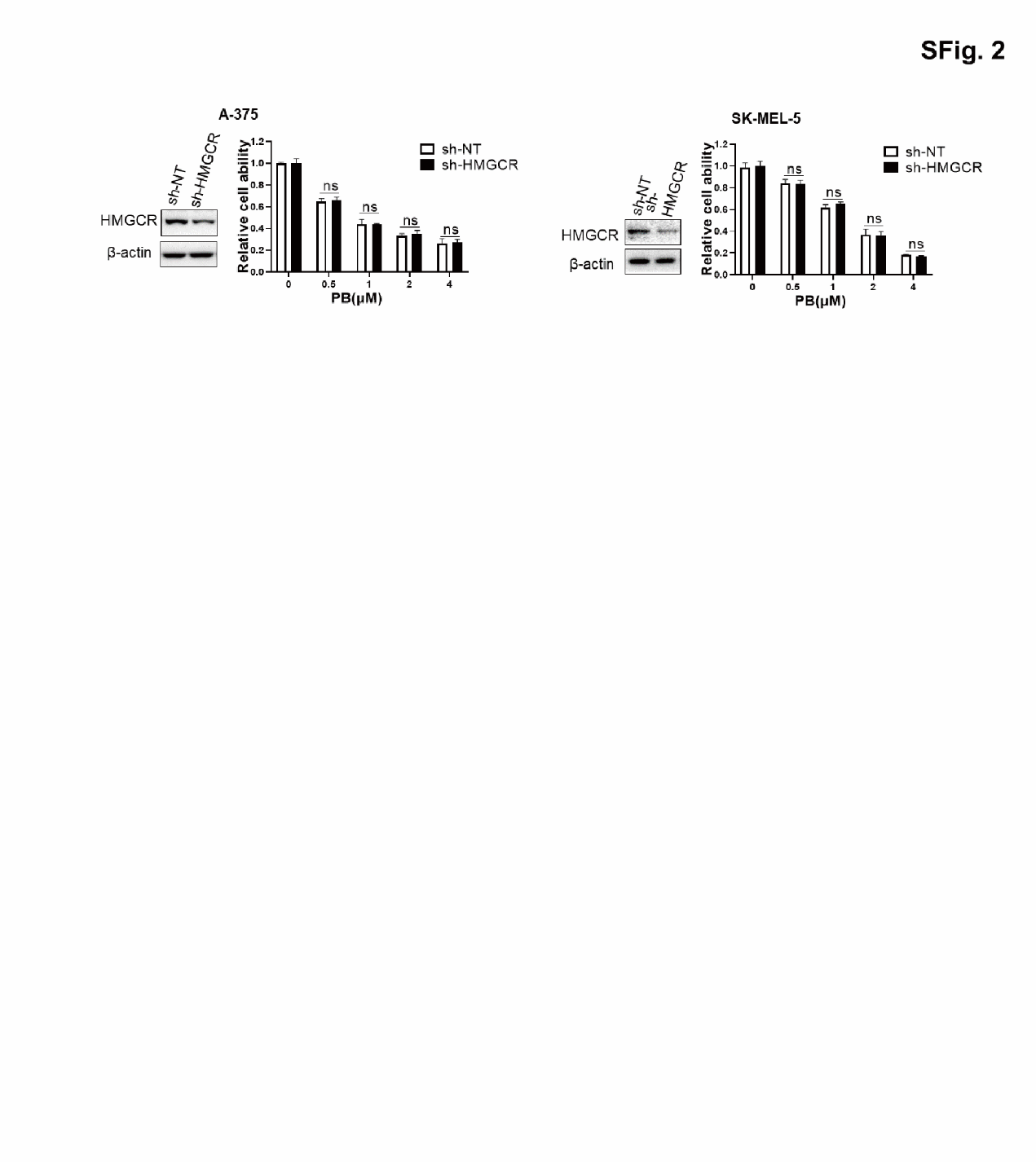
**

**Supplementary figure 2 The effects of HMGCR knockdown on the proliferation of melanoma cells treated with PB.** The A-375 and SK-MEL-5 cells were treated with the indicated concentrations of PB for 72 h with or without HMGCR knockdown, and the viability was examined by CCK8 assay. The level of HMGCR protein was examined by Western blotting. β-actin serves as a loading control.

**
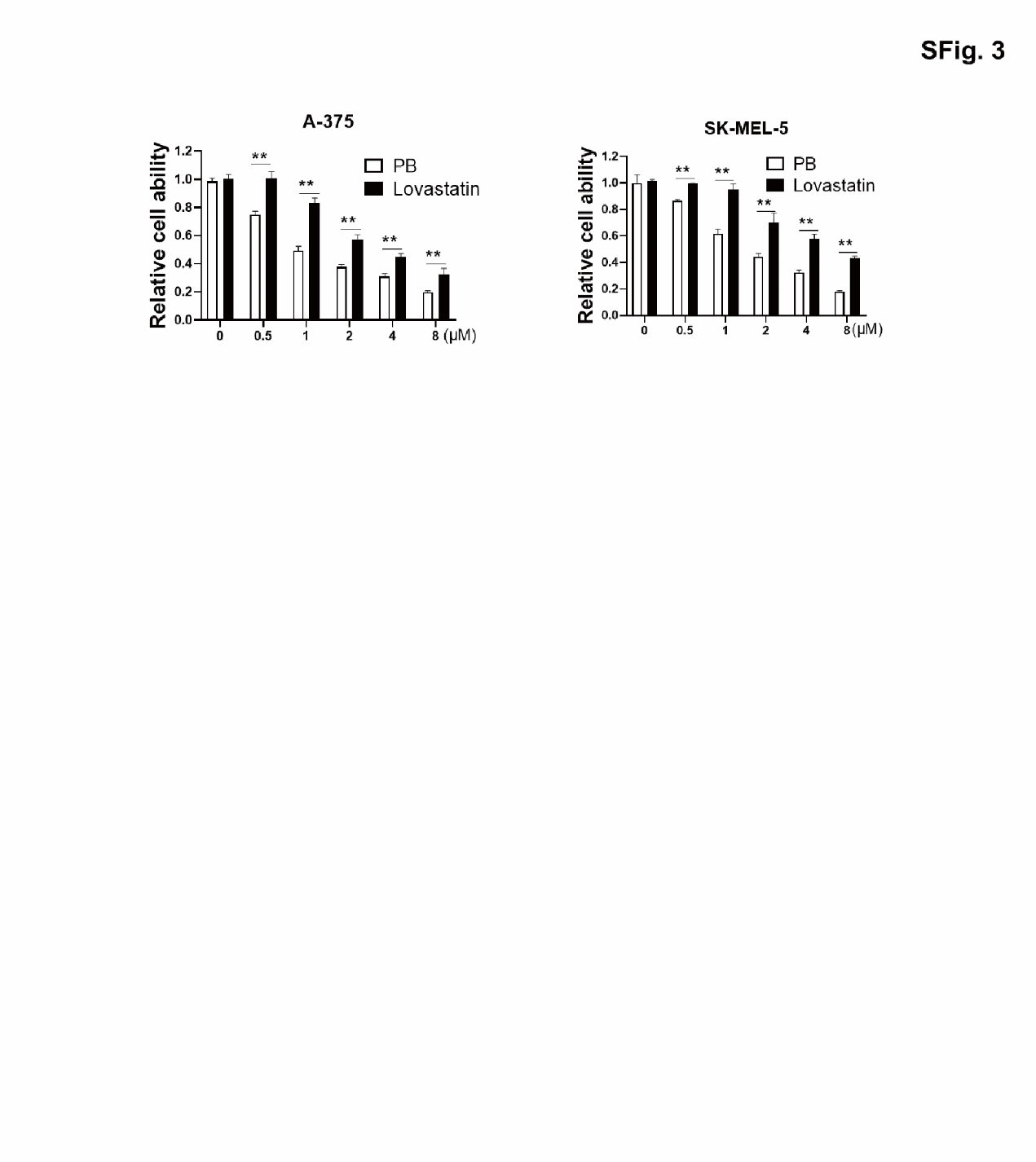
**

**Supplementary figure 3 The effects of PB and lovastatin on the proliferation of melanoma cells.** The A-375 and SK-MEL-5 cells were treated with the indicated concentrations of PB or lovastatin for 72 h, and the cell viability was determined by CCK8 reagent.

**
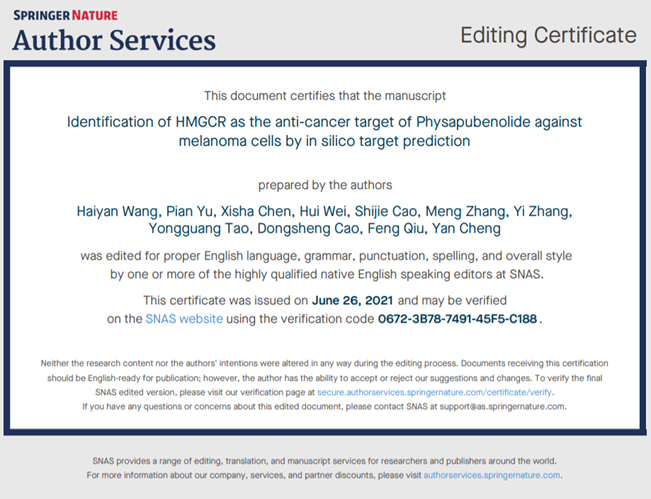
**

**Supplementary figure A certificate for such an editing service**

**STable 1.** Other Five potential medicinal targets

| Uniprot_ID | Gene | | Protein | SAR_ Consensus |
| --- | --- | --- | --- | --- |
| P04035 | HMGCR | 3-hydroxy-3-methylglutaryl-coenzyme A reductase | | 0.942 |
| P05093 | CYP17A1 | Steroid 17-alpha-hydroxylase/17,20 lyase | | 0.203 |
| P10275 | AR | Androgen receptor | | 0.413 |
| P30556 | AGTR1 | Type-1 angiotensin II receptor | | 0.077 |
| P18031 | PTPN1 | Tyrosine-protein phosphatase non-receptor type 1 | | 0.181 |
| P28845 | HSD11B1 | Corticosteroid 11-beta-dehydrogenase isozyme 1 | | 0.365 |
